# “It’s up to me”: the experience of patients at high risk of cardiovascular disease of lifestyle change

**DOI:** 10.1080/02813432.2020.1794414

**Published:** 2020-07-17

**Authors:** Lena Lönnberg, Mattias Damberg, Åsa Revenäs

**Affiliations:** a Center for Clinical Research, County of Västmanland, Uppsala University, Västerås, Sweden; b Department of Public Health and Caring Sciences; Family Medicine and Preventive Medicine, Uppsala University, Uppsala, Sweden; c School of Health, Care and Social Welfare, Division of Physiotherapy, Mälardalen University, Västerås, Sweden

**Keywords:** Qualitative research, diabetes mellitus, type 2, hypertension, primary care, counselling, lifestyle change

## Abstract

**Objective:**

Despite knowledge of the effect of lifestyle changes in preventing cardiovascular disease, a large proportion of people have unhealthy lifestyle habits. The aim of our study is a) to explore the experiences of participants at high risk of CVD of lifestyle change after participation in a one-year structured lifestyle counselling programme and b) to link the techniques and strategies used by the participants to the processes of the transtheoretical model of behaviour change (TTM).

**Design:**

A qualitative explorative design was used to collect data on participants’ experiences. An abductive content analysis was conducted using the processes within TTM for the deductive analysis.

**Setting:**

Patients that participated in a one-year lifestyle counselling programme in Swedish primary care, were interviewed.

**Subjects:**

Eight men and eight women, aged 51–75 years, diagnosed with hypertension or type 2 diabetes mellitus.

**Main outcome measures:**

Experiences of lifestyle change in patients at high cardiovascular risk.

**Results:**

The analysis yielded four dimensions that assisted lifestyle change: ‘The value of knowledge’, ‘Taking control’, ‘Gaining trust in oneself’ and ‘Living with a chronic condition’. The theme ‘It’s up to me’ illustrated that lifestyle change was a personal matter and responsibility.

**Conclusion:**

Enhanced knowledge, self-efficacy, support from others and the individual’s insight that it was his/her own decisions and actions that mattered were core factors to adopt healthier lifestyle habits.

**Practice Implications:** Although lifestyle change is a personal matter, the support provided by primary healthcare professionals and significant others is essential to increase self-efficacy and motivate lifestyle change.

**Key Points**
- A large proportion of people persist to have unhealthy lifestyle habits also after receiving a diagnosis of hypertension or diabetes mellitus, type 2.
- This study contributes to enhanced knowledge of how patients experience lifestyle change after counselling in primary care.
- Both experiential and behavioural processes as defined by the transtheoretical model of behaviour change were used to make lifestyle changes by the patients in this study.

## Introduction

Although there have been substantial improvements in cardiovascular disease (CVD) outcomes, non-communicable diseases—including ischemic heart disease, type 2 diabetes mellitus (T2DM), stroke and chronic obstructive pulmonary disease—still account for the majority of deaths and disability-adjusted life-years (DALYs) worldwide [1]. The leading underlying risk factor in 2017 for death and DALYs was high systolic blood pressure followed by smoking, high fasting plasma glucose, high body-mass index and high levels of low-density lipoprotein cholesterol, which are factors in which lifestyle habits play a crucial role [2].

Guidelines from the American Heart Association, European Heart Association as well as the Swedish National Board of Health and Welfare emphasize that the highest clinical priority for CVD prevention should be given to individuals with prevalent CVD or those at high risk of developing CVD [2–4]. Despite the guidelines and the knowledge of the effect of lifestyle changes in preventing future CVD through practices such as improved diet, increased physical activity (PA) and smoking cessation, a large proportion of individuals at high risk of CVD do not comply [5–7]. A team-based, patient-centred approach that addresses all aspects of a patient´s lifestyle habits has previously been proposed as an effective strategy for CVD prevention in clinical practice [4,8,9]. However, scientific evaluations of lifestyle programmes in primary care are scarce, and structured lifestyle counselling is still not integrated into everyday clinical practice [4,8,9]. To improve the care of individuals at high risk of CVD, a structured lifestyle programme was launched at a primary care unit in Västerås, Sweden. The programme has previously been described by Lonnberg et al. [10]. To improve the effectiveness of lifestyle interventions, it is important to investigate how patients with chronic illness manage lifestyle changes after diagnoses of conditions such as T2DM or hypertension, and thereby prevent future CVD.

Different models have been used to understand how people make lifestyle changes e.g. the transtheoretical model of behaviour change (TTM)[11], social cognitive theory [12,13] the theory of planned behaviour [14] and the self-determination theory [15]. One common concept for all these theories are the construct of self-efficacy. How the individuals are able to perform lifestyle changes are linked to his/her perception of whether he/she is capable of pursue these changes. Another often used term in preventive health care is ‘empowerment’, a concept frequently used in diabetes care and health promotion. According to the World Health Organisation empowerment stands for a process where people see a closer correspondence between their goals in life and a sense of how to achieve them, and a relationship between their efforts and life outcomes [16].

The transtheoretical model of behaviour change was first presented by James Prochaska and Carlo di Clemente in the field of smoking cessation [11] and has been successfully applied to a variety of interventions targeting different health-related behaviours such as smoking, alcohol addiction, dietary habits and drug addiction. Although TTM is commonly used there are some studies that has implied that the processes involved differ depending on the target habit, e.g. physical activity and diet [17–19]. Considering these incongruences, it would be interesting to enhance the understanding of this area and add to previous knowledge of the TTM.

Thus, the aim of our study is a) to explore the experiences of participants at high risk of CVD of lifestyle change after participation in a one-year structured lifestyle counselling programme and b) to link the techniques and strategies used by the participants to the processes of change in the TTM.

## Material and methods

### Design

A qualitative content analysis with an explorative design was performed [20,21] to describe the participants’ experiences of lifestyle change. The abductive analysis method was used, which has a deductive step using the ten processes of change, followed by an inductive step [22]. Data were collected from 16 semi-structured, individual interviews of people who participated in a one-year lifestyle programme in a primary care unit in Västerås, Sweden during 2015. The lifestyle programme was delivered by one of four district nurses and have been described in detail earlier in a previous publication [10]. This study was approved by the local ethics committee at Uppsala (DNR 2014/497/1) and all informants provided informed written consent.

### The transtheoretical model of behaviour change

The transtheoretical model of behaviour change is an integrative theory of therapies to enhance an individual’s readiness to act on a healthier behaviour. The model comprehends one temporal dimension of behavioural change more known as ‘The stages of change’. Change is described as a process involving progress through a series of stages i.e. ‘precontemplation’, ‘contemplation’, ‘preparation’, ‘action’ and ‘maintenance’. An individual uses different strategies or processes to progress through the stages. The processes focus on *how* change is made, i.e. the strategies and techniques used to change behaviour. The ten processes that will be used in the deductive part of our analysis are presented in Table 1 [17].

**Table 1.** The Processes of change within the Transtheoretical model of behaviour change (Prochaska et al. [11]).

Experiential	Behavioural
Consciousness raising	Helping relationship
*(increasing awareness)*	*(support for the healthy behaviour change)*
Dramatic relief	Counter conditioning
*(emotional arousal)*	*(substituting undesired behaviour)*
Self-reevvaluation	Stimulus control
*(self-reappraisal)*	*(avoidance, environmental re-evaluation)*
Environmental re-evaluation	Reinforcement management
*(social reappraisal)*	*(overt or covert reinforcement)*
Social liberation	Self-liberation
*(environmental opportunities)*	*(committing to act)*

### Participants and recruitment

We used a purposive sampling from the population of individuals who completed the one-year lifestyle programme in 2015. Inclusion criteria were the following: diagnosis (hypertension, T2DM or impaired glucose tolerance), aged 30 to 75 years old, fluent in Swedish and having had counselling sessions over one year with one of four district nurses in order to represent different ages, gender, diagnoses and having met any of the four district nurses. Eligible individuals were contacted during May 2018 by the lifestyle nurses and asked to consent to an interview with the first author, LL. To gather the richest data possible, we aimed to recruit a variety of individuals in terms of diagnosis, sex, age and information on which of the four nurses they met. LL contacted possible informants, introduced them to the study and scheduled an interview with those who consented. All but one agreed to participate (owing to lack of time).

A total of 16 individuals consented to participate, Table 2. All informants had made lifestyle changes regarding PA and dietary habits, and one informant also cut down on daily smoking. The informants have been given pseudonyms to protect their privacy and to enrich the result presentation by making it more authentic.

**Table 2.** Background information of the informants: sex, diagnose, birth year, attending nurse (A–D), and alias.

Informant	Sex	Diagnose	Birth year	Nurse (A-D)	Alias
1	female	HT	−44	A	Lisa
2	male	T2DM	−48	C	Pelle
3	female	T2DM	−43	D	Inger
4	female	HT	−43	A	Maria
5	male	HT	−46	B	Olof
6	male	T2DM	−61	A	Anders
7	female	HT	−51	B	Anna
8	female	T2DM	−45	C	Tove
9	male	HT	−63	A	Sven
10	female	IGT	−50	C	Malin
11	male	T2DM	−67	C	Fredrik
12	female	HT	−51	D	Eva
14	female	HT	−63	B	Åsa
13	male	T2DM	−56	C	Gunnar
15	male	HT	−60	D	Lars
16	male	HT	−51	B	Nils

HT: hypertension; T2DM: type 2 diabetes mellitus; IGT: impaired glucose tolerance.

### Data collection

The interviews took place between July and November 2018 at the primary care unit where the lifestyle programme was conducted. Each interview lasted between 25 and 40 min. The interviews were recorded using a Philips Digital Pocket Memo DPM 8000/00 and were transcribed verbatim by LL.

The semi-structured interviews followed a guide prepared by LL (physiotherapist in primary healthcare, PhD student) and the last author, ÅR (physiotherapist and researcher), with two main questions in mind: (1) What was the participants’ experience of lifestyle change? and (2) What was the participants’ experience of lifestyle counselling? Every question was followed up with prompts like ‘Can you tell me more?’ or ‘Can you give me some more examples?’. The interview guide was piloted with two participants. This resulted in a slight modification to the guide (an additional summary of the lifestyle programme as an introduction). After an additional 10 interviews, the information started to repeat and after four more interviews, the information collected (including the two test interviews) was considered rich enough to answer the research questions.

### Data analysis

To explore the experience of individuals at high risk of CVD of lifestyle change, a qualitative content analysis was performed. An abductive approach was used that entailed moving back and forth between inductive and deductive analyses [20–22].

After the interviews had been transcribed, they were re-read several times to obtain an overview and an overall sense of the material. As a *first* step, meaning units were identified; texts concerning information about the informants’ experiences of lifestyle change were highlighted and put into a matrix for further condensation. In the *second* step, all condensed meaning units were coded and then analysed in a deductive manner by sorting the codes according to the processes of change. As a *third* step, the codes were sorted into subcategories based on similar manifest content in an inductive manner. Thereafter, as a *fourth* step, subcategories were sorted into categories reflecting their content. After categorization of all codes, we went back to the deductive analysis to ensure that the codes and categories were accurate and related to the processes of change. In the *final* step, a theme indicating an interpretation of the text emerged from latent content. For an example of the analysis from meaning unit to category see Table 3.

**Table 3.** Example from the analysis of transforming meaning units to condensed meaning units, codes, subcategories and categories.

Pat int nr/ page/row	Meaning unit	Condensed meaning unit	Code	Subcategory	Category
1/6/22-24	Well, it is my knees that constrains me a little bit. But as long as I walk straight forward [laugh] and not a lot of… well, and I don´t dare to run any more. Sometimes I run to the bus, but that is not so good.	The knees constrain, but if I walk straight forward and not run it´s ok.	Modify physical activity so it works with physical impairment	Modifying physical activity to my physical condition	Taking control
14/4/31-32	And then I realized that the blood pressure was much lower, so I go there and try to check it regularly	Checks blood pressure regularly	Monitor your values/ test results	Test results is motivating	The value of knowledge

### Trustworthiness

Transcription of the interviews were done by LL shortly after the interviews had been performed. Checking the transcripts against all audio files were done at two occasions by LL, and for two interviews also by ÅR. The analysis was mainly performed by LL and discussed several times with ÅR to increase its trustworthiness. The two test interviews were included in the study after careful consideration. The authors did not consider that the addition of a short recall of the lifestyle programme had a severe impact on their response as opposed to the other interviews. To start, the first two interviews were analysed separately regarding meaning units by both authors and then discussed to provide coherence. The remaining interviews were analysed by LL. The coding and sorting according to the process of change and labelling of the inductive categories were discussed by the two authors to refine the analysis, for example by re-labelling codes and categories and re-sorting some of the codes and subcategories. The results were also considered by a third researcher experienced in qualitative research who had not previously been involved in the project.

## Results

The analysis resulted in 148 codes, belonging to eight of the ten processes of change described in the TTM, 18 subcategories, four categories and one theme. The categories reflected the manifest content in four dimensions that all contributed to the individual’s ability to make a lifestyle change. They played different roles in experiences of lifestyle change. The participants described a personal responsibility and role in lifestyle change that informed four categories: ‘The value of knowledge’, ‘Taking control’, ‘Gaining trust in oneself’ and ‘Living with a chronic condition’.

**Figure 1.**
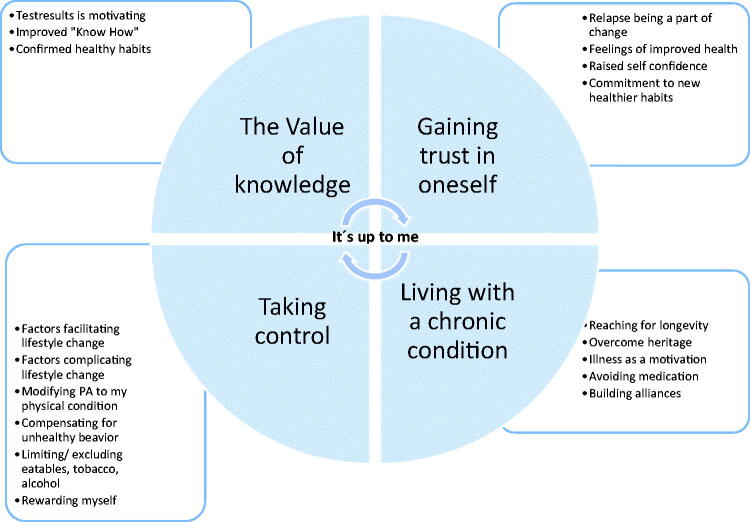
Overview of the result presenting the theme “It´s up to me” and how the four categories and subcategories are connected.

The theme ‘It’s up to me’ interpreted the latent content and emphasized the importance of the person’s own actions in lifestyle changes. For an overview of the results, see Figure 1 and Table 4.

**Table 4.** Codes, subchategories and chategories sorted by processes within the transtheoretical model.

Experiental	Code	Subcategory	Category
***Consciousness raising***	*Likes to follow measurements*	Test results are motivating	**The value of knowledge**
	*Wants to see better results*	“	“
	*Test results motivated increased PA*	“	“
	*Test results motivated lifestyle changes*	“	“
	*Needed to see results from test*	“	“
	*Oxygen uptake was lower than before*	“	“
	*Monitor values/ test results*	“	“
	*Use scale regularly*	“	“
	*Use blood pressure monitor at home regularly*	“	“
	*Learn how body responds*	“	“
	*Decreased WC/blood pressure after regular PA*	“	“
	*Try to reduce stress*	Improved “Know how”	**The value of knowledge**
	*Eats more vegetables because they are healthy*	“	“
	*Nothing wrong with fewer sweets*	“	“
	*Making healthy sandwiches*	“	“
	*Received brochures with healthy recipes*	“	“
	*Read about my illness on internet*	“	“
	*Information from different sources*	“	“
	*Need to know how to deal with low motivation*	“	“
	*Information about PA*	“	“
	*Information about illness and lifestyle*	“	“
	*Know what to eat or not to eat*	“	“
	*Received information about small changes*	“	“
	*Tested and learned about new ingredients*	“	“
	*The information should be more frightening*	“	“
	*Learned how to breathe*	“	“
	*Shared knowledge with others*	Confirmed healthy lifestyle habits	**The value of knowledge**
	*Already have healthy eating habits*	“	“
	*Already have healthy PA habits*	“	“
	*Already changed lifestyle habits*	“	“
	*Do not smoke or drink*	“	“
***Dramatic relief***	*Staying healthy*	Reaching for longevity	**Living with a chronic condition**
	*Want to see grandchildren grow up*	“	
	*Do not want the same fate as relatives*	Overcome heritage	“
	*Fear of deterioration of illness*	Illness as a motivation	“
	*Trying to stop the progress of diabetes*	“	“
	*Diagnosis motivates lifestyle change*	“	“
	*Cannot cheat if one has DMT2*	“	“
	*Do not want medicine*	Avoiding medication	“
	*Lifestyle change instead of medicine*	“	“
	*Fear of having to take injections*	“	“
***Self re-evaluation***	*Less healthy over time*	Relapses are part of change	**Gaining trust in oneself**
	*Continue to smoke*	“	“
	*Increased smoking after end of lifestyle programme*	“	“
	*Decreased PA over time*	“	“
	*Less healthy eating habits over time*	“	“
	*Weight gain over time*	“	“
	*Easy to return to bad habits*	“	“
	*Avoid cigarettes to prevent relapse*	“	“
	*Need to compensate after vacation for food habits*	“	“
	*Keep on thinking of healthy food habits*	“	“
	*Eat unhealthily in the evenings*	“	“
	*Stress has a bad influence on lifestyle habits*	“	“
	*Stress exacerbates illness*	“	“
***Self re-evaluation***	*Pain makes PA difficult*	Factors complicating lifestyle change	**Taking control**
	*Stress leads to wrong decisions*	“	“
***Self re-evaluation***	*Lost interest when PA became competitive*	“	**Taking control**
	*Difficult to stay motivated*	“	“
	*Weather makes PA difficult*	“	
	*Eats unhealthily on the weekends*	“	“
	*Relatives affect decisions*	“	“
	*Lack of supervision*	“	“
	*Relatives prepare unhealthy food*	“	“
	*Gradual change of lifestyle*	Factors facilitating lifestyle change	“
	*More time for PA after retirement*	“	“
	*PA should be fun and at your own pace*	“	“
	*PA should be close to your home*	“	“
	*Choose the PA you like*	“	“
	*Medication can be a part of change*	“	“
	*Follow a daily routine*	“	“
	*Prioritize PA over something else*	“	“
	*A dog makes you walk every day*	“	“
	*PA together with others*	“	“
	*PA as a daily routine*	“	“
	*It is easier to avoid foreign sweets*	“	“
	*Choose the right time for PA*	“	“

Behavioural	Code	Subcategory	Category
***Counter conditioning***	*Use indoor cycling when weather is bad*	Factors facilitating lifestyle change	**Taking control**
	*Working out can include different activities*	“	*“*
	*Bicycling instead of walking*	“	*“*
	*Brain workout*	“	*“*
	*Time spent at summer home enables PA*	“	*“*
	*Taking care of garden and house entails PA*	“	*“*
	*Increased intensity of PA*	“	*“*
	*Work tasks can imply PA*	“	*“*
	*Alternated between activities*	“	*“*
	*Worked out despite pain*	Modifying PA to my physical condition	“
	*Avoid specific gym machines*	“	“
	*Choose activity that works with physical impairment*	“	“
	*Start slow and easy*	“	“
	*Modify PA so it works with physical impairment*	“	“
	*Add PA when blood sugar is high*	Compensating for unhealthy behaviour	**Taking control**
	*Add PA after bad eating day*	“	“
	*Add PA to compensate smoking*	“	“
	*Working out not to alleviate conscience*	“	“
	*Walking to compensate for prolonged sitting*	“	“
***Helping relationship***	*Shared task when cooking together*	Building alliances	**Living with a chronic condition**
	*Shared knowledge with spouse*	“	“
	*PA is easier together with spouse*	“	“
	*Spouse can also benefit from lifestyle change*	“	“
	*Stopped smoking with spouse*	“	“
	*Spouse makes healthy food*	“	“
	*Want to do as the nurse says*	“	“
	*Joint goal with nurse*	“	“
	*Beneficial to meet the same nurse*	“	“
	*Good to have someone to talk to*	“	“
	*Recurrent meetings were motivating*	“	“
	*Easier to adhere to healthy habits when monitored by nurse*	“	“
***Helping relationship***	*Behaved well to avoid being banned by the nurse*	“	**Living with a chronic condition**
	*Pep talk from nurse was motivating*	“	“
	*Shared results with other health care workers*	“	“
***Stimulus control***	*Cut down on sweetened food/cold cuts*	Limiting/ excluding ingredients, tobacco and alcohol	**Taking control**
	*Need to be more conscious about food*	“	“
	*Cut down on portion size*	“	“
	*Avoid buffets*	“	“
	*Cut down intake of sweets*	“	“
	*Cut down on potatoes and pasta*	“	“
	*Only drink wine on weekends*	“	“
	*Stopped eating sweets*	“	“
	*Only eat healthy food*	“	“
	*Eat fewer carbohydrates when on diet*	“	“
	*Cut down on smoking*	“	“
	*Want to be able to eat the same food as others*	“	“
	*Eat more vegetables*	“	“
	*Eat crispbread if hungry between meals*	“	“
	*Eat more vegetables than before*	“	“
	*New kitchen appliance*	“	“
	*Meal replacements instead of lunch*	“	“
	*Changed to drinking water at all meals*	“	“
	*High quality sausage as alternative*	“	“
	*Better to stop smoking altogether, not try to cut down*	“	
***Reinforcement management***	*No need for medication after lifestyle change*	Feelings of improved health	**Gaining trust in oneself**
	*Better endurance after regular PA*	“	“
	*Better joint mobility after regular PA*	“	“
	*PA makes you less depressed*	“	“
	*Good feeling after work out*	“	“
	*Celebrate when goal is achieved*	Rewarding myself	**Taking control**
***Self liberation***	*Make my own decisions*	Raised self confidence	**Gaining trust in oneself**
	*Share knowledge with others*	“	“
	*Cannot wait for improvement to come by itself*	“	“
	*Self-awareness*	“	“
	*Time is my own priority*	“	“
***Self liberation***	*Goal motivates lifestyle change*	Commitment to new, healthier habits	
	*A promise shall be kept*	*“*	**Gaining trust in oneself**
	*Goal motivates lifestyle change*	“	“
	*Make decision to improve unhealthy habit*	“	“
	*Maintain Improved healthy habits*	“	“

### The value of knowledge

This category, with its three subcategories, related to one experiential process—consciousness raising. The informants expressed the view that changes to a healthier lifestyle require knowledge, motivation and a sense of awareness of how choices in everyday life affect a person’s condition.

*‘*Test results are motivating*’* described the informants’ experiences of how the knowledge of health measurements and following them over time supported lifestyle change. They reported that knowledge about test results regarding for example blood samples and anthropometric measurements was both motivating and encouraging. This was also said to allow the individual to connect certain behaviours to a specific physical effect, which was described as motivating.

I like to see my blood pressure drop, or weight. And my results from the bicycle test! That was the best, I think! (Lisa, high blood pressure)

*‘*Improved know-how*’* described the informants’ expressions that knowledge about subjects such as food, PA and medication increased their motivation to make lifestyle changes. Moreover, suggestions on small changes to enhance PA and eating habits were found to be useful by the informants.

*‘*Confirmation of lifestyle habits’ explained how having healthy habits confirmed by the nurse was reported to be helpful and guided the informant on which lifestyle habit needed to be addressed and which was already sufficient.

### Taking control

Taking control included six subcategories related to both experiential (self re-evaluation) and behavioural processes (counter-conditioning, reinforcement and stimulus control). The category was based on a description of the informants’ awareness of what factors facilitated or complicated lifestyle changes as well as more behavioural processes. All six subcategories were expressions of more active, overt actions by the individual to make lifestyle changes.

‘Factors facilitating lifestyle change’ described the informants’ statements about factors that made lifestyle changes easier, such as choosing an activity they liked, geographic proximity to home or workplace and finding a suitable time and day. In addition, retirement could free more time to engage in PA and adopt healthier eating habits.

‘Factors complicating lifestyle change’ were obstacles to lifestyle change, such as shortage of time, stress, bad weather and seasonal changes. Moreover, close relatives could be a barrier, for example when they did not want to share the same food or when they prepared unhealthy food that was not in line with new, healthier eating habits. Another barrier was when the informant did not feel ill.

I don’t *feel* sick any way. I think that is a part of the problem why it is so hard to change eating habits. (Fredrik, T2DM)

‘Modifying PA to my physical condition’ referred to the informants’ descriptions of coping with physical impairment and finding new ways to be physically active. The informants described different ways of managing problems, such as joint problems or back pain, by both revising and modifying intensity, activity of choice and duration of PA.

‘Compensating for unhealthy behaviour’ by being more physically active after eating badly one day was said to be one way of compensating for lapses. Living as healthily as possible in every other way was also described as compensation for continuing to smoke.

‘Limiting/excluding ingredients, tobacco or alcohol’ referred to the informants’ different ways of addressing habits they considered to be less healthy. This could be expressed as avoiding certain ingredients such as sugar or carbohydrates or starting to eat according to the ‘plate model’ (i.e. half the plate filled with vegetables and the other half filled with equal parts of carbohydrates and protein) or going to an à la carte restaurant instead of a buffet.

‘Rewarding myself’ referred to the informants’ descriptions of using external rewards for goal achievement, such as having a party when their waist circumference fell below a certain level.

### Gaining trust in oneself

This category contained four subcategories belonging to both experiential (self re-evaluation) and behavioural processes (re-enforcement management and self-liberation). The informants reported the use of different processes to change their lifestyles even in the later action-oriented stages of change. They exposed more covert phenomena of feelings and thoughts within the informant that led to lifestyle changes.

‘Relapse as part of change’ referred to the informants’ statements about their awareness of changes in behaviour, sometimes relapsing to former (unhealthier) habits and possible explanations for these. The informants expressed awareness of events, such as a decline in PA or weight gain after the counselling sessions were over. However, they also described a desire to be active again, with or without support from others. Reflecting upon changes over time could raise motivation to re-engage in prior healthier behaviours.

‘Feelings of improved health’ were the reinforcements that informants expressed when they felt physically and psychologically good after being physically active.

I can feel my physical condition improving when I walk; my legs and I feel better. (Tove, T2DM)

‘Raised self-confidence’ referred to declarations that the participants gained confidence in their ability to change their lifestyle habits and the importance of this for addressing lifestyle changes. They reported new insights into their own decisions and the awareness that prioritizing their actions was in their own hands.

‘Commitment to new healthier habits’ was the informants’ expressed desire to take charge of their lifestyle habits. Setting goals and making promises to themselves (not the nurse or spouse) were tools to make those lifestyle changes and recognize that they had to make their own decisions. As one informant stated:

I like cookies and buns, and my partner likes to bake and hasn’t understood that I shouldn’t eat them, but I have to make my own decisions. (Pelle, T2DM)

### Living with a chronic condition

Four of the subcategories included in this category related to an experiential process (dramatic relief) and one to a behavioural process (helping relationships). In combination, they described factors surrounding and supporting the individuals’ lifestyle changes and described the informants’ experiences of the ways in which they manage their diagnoses and its impact on lifestyle change.

‘Reaching for longevity’ was expressed as the reason for the informants to improve their eating and PA habits. The informants enunciated a wish to play with their grandchildren and to spend more quality time with their loved ones as motivation for lifestyle change.

You want to live a little longer, to stay healthy… If you want to see your grandchildren grow up, then you have to keep up. (Eva, high blood pressure)

‘Overcome heritage’ represented the informants’ expressed fears of meeting the same destiny as their parents or close relatives regarding disease. This was recognized as part of why they believed lifestyle change to be important.

‘Illness as a motivator’ was the informants’ report that the illness itself could be a motivating factor, for example a desire to avoid progressing from impaired glucose tolerance to T2DM.

‘Avoiding medication’ was also reported as a motivational factor by the informants. This could be expressed as not wanting to take blood pressure lowering medication or for example, to avoid the need for injected medicines:

I’ll better fix this, or else there will be syringes. That was an eye-opener! (Anders, T2DM)

‘Building alliances’ referred to the informants’ experiences of the importance of support from others to make lifestyle changes. This could include a spouse, a friend or a nurse. The informants reported that the feeling of being ‘supervised’ provided guidance in making healthier choices. The counselling sessions with the nurse were described as giving the informants the opportunity to reflect on current and future lifestyle habits.

## Discussion and conclusion

### Statement of principal findings

The results of the qualitative analysis revealed four dimensions that described the experiences of people at high risk of CVD of lifestyle change: ‘The value of knowledge’, ‘Taking control’, ‘Gaining trust in oneself’ and ‘Living with a chronic condition’. The results highlighted the importance of being knowledgeable about one’s condition, including monitoring and providing feedback on health parameters motivating the individuals to take control and act to enhance their well-being. Support from others, identifying facilitating and complicating factors and gaining self-confidence in making lifestyle changes were also described as fundamental. The theme of ‘It’s up to me’ illustrated the core of lifestyle changes as a personal matter and responsibility.

### Discussion

Both ‘The value of knowledge’ and ‘Taking control’ underline the importance of in-depth knowledge about diagnoses and possible outcomes as well as an enhanced awareness of various measures. This is confirmed by another qualitative study concluding that the diagnosis of pre-diabetes itself motivated the participants to be more conscious of what they ate and to increase their PA to avoid progressing from pre-diabetes to diabetes [23]. On the other hand, previous studies have also shown that not every individual wants to be informed about their future risk connected to a chronic condition [24]_._ Therefore, it is essential to meet the individuals’ personal needs to optimize their counselling regarding behaviour change to manage a chronic condition [24,25].

The identification of facilitating and complicating factors for lifestyle change was described as crucial by the informants. This is consistent with other qualitative studies regarding changes in dietary habits and PA. For example, a Finnish study [26] with 74 subjects at high risk of T2DM showed similar experiences of facilitating factors for PA as our study, for example, enjoyment, social relationships and benefits to health and encouragement from others. Barriers noted were weather, season, health problems and lack of time [26]. Modifying PA to one’s present physical condition in our study was also found to be important, and the result may indicate that knowledge of ways to address temporary deficits in one’s current physical condition and to recognize different PA alternatives raises self-confidence.

The ‘Gaining trust in oneself’ category shares common characteristics with the concept of self-efficacy. Self-efficacy is a core concept of social cognitive theory whereby belief in personal capacity plays a central role in personal change and is founded on an agentic perspective [12,13]. The agentic perspective highlights the individual as an agent who intentionally make things happen by his/her own action [12].

Social cognitive theory distinguishes between three modes of agency: personal, proxy and collective agency. Personal agency is about setting health goals, making concrete plans and realizing them, and it depends on the individual’s self-efficacy beliefs. The most effective way of creating a strong sense of self-efficacy is either by studying others or through mastery experiences [12,13]. This accords with the statements by our informants that setting their own achievable goals was important for commitment and making their own decisions about lifestyle change.

The concept of self-efficacy is present in several other behaviour theories, such as TTM [11] the theory of planned behaviour [14] and the self-determination theory [15], although, sometime, it is described in different terms. It seems that this core dimension, which could be described as capacity, perceived behavioural control, competence or self-confidence, is an important universal quality of human behaviour for our personal agency.

‘Empowerment’ is another concept used in the field of health promotion and encompasses actions directed at strengthening skills and capacities of the individual in order to master his/her condition [16]. This concept could also be applicable for the care of patients with T2DM or hypertension in our study, as setting goals, both regarding enhanced health behaviour and treatment were essential in the lifestyle programme. Personal agency has a close relationship also to empowerment as it encourages the individual to exert agency when he/she influence or make decisions about their health care. An interesting qualitative study by Hultberg *et al* enlightens patient agency through resistance in decision-making about cardiovascular preventive drugs. One of their findings was that the recognition of active or passive resistance is valuable for a shared decision-making and that it supports personal agency [27].

Our results indicate that involvement of others in lifestyle changes can be both encouraging and aggravating when a person is living with a chronic condition. This was also revealed in a Swedish interview study with 10 informants diagnosed with T2DM who expressed ambiguous feelings about close relatives or friends who interfered with the informants’ behaviour. Sometimes the actions of significant others can even provide an excuse not to adhere to a healthier lifestyle [28]. The alliance with the nurse was pointed out by our study informants as an important motivation for lifestyle change as well as a source of confirmation that they were on the right track in the process of change. Being confident in the nurse’s ability to provide support for lifestyle change, without judging, has previously been described as essential and necessary in facilitating a variety of lifestyle changes, such as smoking cessation, diet and PA [29].

In our study, we used the processes of the TTM to perform the deductive analysis of the interviews. The informants reported that they had undertaken several actions to address previous unhealthy habits. Therefore, we can assume that they were at either the ‘action’ or ‘maintenance’ phases. Because all the informants had made lifestyle changes, it is not surprising that the results were related to both experiential and behavioural processes. According to a meta-analysis of the TTM and its application to PA behaviour change, experiential processes tend to peak during action and behavioural processes peak in maintenance. On the other hand, the study also concludes that the distinction between experiential and behavioural construct may not be applicable to understand how the processes are used to achieve PA behaviour change, suggesting that TTM offers limited explanation for changes in PA habits [18]. In our study, two of the processes (environmental re-evaluation and social liberation) were not related to the data. That contrasts with a 48-week randomized controlled trial where 48 women participated in a study to increase PA with the TTM as basis for behavioural counselling. According to that study, all 10 processes were present at both baseline and at follow-up at 48 weeks [30]. One explanation for the two processes not being present in our study could be that the lifestyle programme was based on a motivational interviewing technique, thereby focusing on the individual’s abilities, priorities and goals. Second, we did not specifically ask the informants about how environmental or social factors might affect their choice of action. If they had been specifically asked about these factors, it is possible that the informants would have expressed thoughts on how the environment and social contexts influenced their behaviour.

### Strengths and limitations

This qualitative study has several strengths. First, we find the informants represent the population that received lifestyle counselling at the primary care unit, representing different ages, gender, diagnoses and having met any of the four district nurses that delivered the counselling. The purposive sampling was not limited by non-responders, in fact all but one agreed to participate. Second, all interviews were performed by the same researcher, ensuring that the same topics from the interview guide were handled in the same way. Third, trustworthiness was addressed by involving several researchers in the analysis process. Finally, the use of TTM and the processes of change enable a solid base for the deductive analysis.

One limitation of this study is that the informants were interviewed three years after completing the lifestyle programme, which could cause ‘recall bias’. Therefore, we started the interviews with a summary of the lifestyle programme. Although impaired memory might alter views of lifestyle change, elapsed time could offer a better perspective of the informants’ experience of lifestyle change. Also, we don´t have information from individuals not participating in the programme. It is likely there are barriers to participate in a lifestyle programme and this would be of interest to explore in further studies. Another limitation is that LL did the deductive coding by herself. Even though the coding was thoroughly discussed with ÅR, the analysis would have been even more reliable if the two authors had coded some of the data separately and then discussed the coding to agree on how to code the data into the processes. Finally, the primary care unit in focus for our study has patients from high socio-economic circumstances, indicated by a low Care Need Index [31], which makes transferability to other social contexts, age groups and nationalities difficult. Despite this, our analysis may support the design of lifestyle counselling for individuals at high risk of CVD in a primary care setting as well as at other outpatient clinics.

### Conclusion

The results from this study have enhanced knowledge of how to coach individuals to make lifestyle changes in a primary care setting. A range of different counselling strategies were required to support lifestyle changes. Enhanced knowledge, self-efficacy, support from others and eventually the individual’s insight that it is his/her own decisions and actions that matter were core features in the motivational process for adopting a healthier lifestyle to minimize the impact of a chronic condition. Experiential processes were frequently used even though the informants were at an action or maintenance stage. The results emphasized lifestyle change as a complex process involving both experiential and behaviour processes that changed over time and differed for everyone.

By exploring the views of lifestyle change from verbal data we strived to add to previous quantitative data of lifestyle change, and more specific to how the processes in TTM was used after participating in a lifestyle programme in primary care. As the clinical consultation was recognized as essential to support lifestyle change and selfcare, it was crucial to hear what people found to be important in order to make acquired changes. This qualitative study of lifestyle change offered a more in-depth knowledge of what really matters, not only in terms of data that can be measured by quantitative data.

### Practice implications

The results imply that lifestyle change is a personal matter; however, the support provided by health care personnel and significant others is essential to increase self-efficacy and motivation to change lifestyles.

## References

[1] Global, regional, and national comparative risk assessment of 84 behavioural, environmental and occupational, and metabolic risks or clusters of risks for 195 countries and territories, 1990–2017: a systematic analysis for the Global Burden of Disease Study 2017. GBD 2017 Risk Factor Collaborators. Lancet. 2018;392:1923–1994. 30496105 10.1016/S0140-6736(18)32225-6 PMC6227755

[2] Arnett DK, Blumenthal RS, Albert MA, et al. 2019 ACC/AHA guideline on the primary prevention of cardiovascular disease. Circulation. 2019;140:596–646. 10.1161/CIR.0000000000000678 PMC7734661 30879355

[3] Kotseva K, Wood D, De Bacquer D, et al. EUROASPIRE IV: A European Society of Cardiology survey on the lifestyle, risk factor and therapeutic management of coronary patients from 24 European countries. Eur J Prev Cardiolog. 2016;23(6):636–648. 10.1177/2047487315569401 25687109

[4] The National Board of Health and Welfare. National guidelines for prevention and treatment of unhealthy lifestyle. Stockholm: The National Board of Health and Welfare; 2018.

[5] The Public Health Agency of Sweden. Nationella folkhälsoenkäten-Hälsapålikavillkor. Stockholm: The Public Health Agency of Sweden; 2017. [Cited 2019 Dec 1]. Available from https://www.folkhalsomyndigheten.se/folkhalsorapportering-statistik/statistikdatabaser-och-visualisering/nationella-folkhalsoenkaten/lenadsvanor/fysisk-aktivitet/2016.

[6] Folsom AR, Yatsuya H, Nettleton JA, et al. Community prevalence of ideal cardiovascular health, by the American Heart Association definition, and relationship with cardiovascular disease incidence. J Am Coll Cardiol. 2011;57(16):1690–1696. 21492767 10.1016/j.jacc.2010.11.041 PMC3093047

[7] Turco JV, Inal-Veith A, Fuster V. Cardiovascular health promotion: an issue that can no longer wait. J Am Coll Cardiol. 2018;72(8):908–913. 30115230 10.1016/j.jacc.2018.07.007

[8] Kotseva K, De Bacquer D, De Backer G, et al. Lifestyle and risk factor management in people at high risk of cardiovascular disease. A report from the European Society of Cardiology European Action on Secondary and Primary Prevention by Intervention to Reduce Events (EUROASPIRE) IV cross-sectional survey in 14 European regions. Eur J Prev Cardiol. 2016; 23:2007–2018. 27638542 10.1177/2047487316667784

[9] Melvin CL, Jefferson MS, Rice LJ, et al. A systematic review of lifestyle counseling for diverse patients in primary care. Prev Med. 2017;100:67–75. 28344120 10.1016/j.ypmed.2017.03.020 PMC6086607

[10] Lonnberg L, Ekblom-Bak E, Damberg M. Improved unhealthy lifestyle habits in patients with high cardiovascular risk: results from a structured lifestyle programme in primary care. Ups J Med Sci. 2019;2:1–11. 10.1080/03009734.2019.1602088 PMC6566702 31063003

[11] Prochaska JO, DiClemente CC, Norcross JC. In search of how people change. Applications to addictive behaviors. Am Psychol. 1992;47(9):1102–1114. 1329589 10.1037//0003-066x.47.9.1102

[12] Bandura A. Social cognitive theory: an agentic perspective. Annu Rev Psychol. 2001; 52:1–26. 11148297 10.1146/annurev.psych.52.1.1

[13] Bandura A. Health promotion by social cognitive means. Health Educ Behav. 2004;31(2):143–164. 15090118 10.1177/1090198104263660

[14] Ajzen I, Madden TJ. Prediction of goal-directed behavior: attitudes, intentions, and perceived behavioral control. J Exp Soc Psychol. 1986;22(5):453–474.

[15] Ryan RM, Deci EL. Self-determination theory and the facilitation of intrinsic motivation, social development, and well-being. Am Psychol. 2000;55(1):68–78. 11392867 10.1037//0003-066x.55.1.68

[16] World Health Organisation. Health improvement glossary. [Internet] Geneva: World Health Organisation; 1998 [Cited 2020 Apr 27]. Available from: https://www.who.int/healthpromotion/about/HPR%20Glossary%201998.pdf.

[17] Marcus BH, Rossi JS, Selby VC, et al. The stages and processes of exercise adoption and maintenance in a worksite sample. Health Psychol. 1992;11(6):386–395. 1286658 10.1037//0278-6133.11.6.386

[18] Marshall SJ, Biddle S. The transtheoretical model of behavior change: a meta-analysis of applications to physical activity and exercise. Ann Behav Med. 2001;23(4):229–246. 11761340 10.1207/S15324796ABM2304_2

[19] Hutchison AJ, Breckon JD, Johnston LH. Physical activity behavior change interventions based on the transtheoretical model: a systematic review. Health Educ Behav. 2009;36(5):829–845. 18607007 10.1177/1090198108318491

[20] Graneheim UH, Lundman B. Qualitative content analysis in nursing research: concepts, procedures and measures to achieve trustworthiness. Nurse Educ Today. 2004;24(2):105–112. 14769454 10.1016/j.nedt.2003.10.001

[21] Graneheim UH, Lindgren B-M, Lundman B. Methodological challenges in qualitative content analysis: a discussion paper. Nurse Educ Today. 2017; 56:29–34. 28651100 10.1016/j.nedt.2017.06.002

[22] Elo S, Kyngas H. The qualitative content analysis process. J Adv Nurs. 2008;62(1):107–115. 18352969 10.1111/j.1365-2648.2007.04569.x

[23] Andersson S, Ekman I, Lindblad U, et al. It's up to me! Experiences of living with pre-diabetes and the increased risk of developing type 2 diabetes mellitus. Prim Care Diabetes. 2008;2(4):187–193. 18996075 10.1016/j.pcd.2008.09.001

[24] Pikkemaat M, Bostrom KB, Strandberg EL. "I have got diabetes!" - interviews of patients newly diagnosed with type 2 diabetes. BMC Endocr Disord. 2019;19(1):53 31126267 10.1186/s12902-019-0380-5 PMC6534850

[25] Kristensen M, Guassora A, Arreskov A, et al. 'I've put diabetes completely on the shelf till the mental stuff is in place'. How patients with doctor-assessed impaired self-care perceive disease, self-care, and support from general practitioners. A qualitative study. Scand J Prim Health Care. 2018;36(3):342–351. 29929420 10.1080/02813432.2018.1487436 PMC6161682

[26] Korkiakangas EE, Alahuhta MA, Husman PM, et al. Motivators and barriers to exercise among adults with a high risk of type 2 diabetes-a qualitative study. Scand J Caring Sci. 2011;25(1):62–69. 20384973 10.1111/j.1471-6712.2010.00791.x

[27] Hultberg J, Rudebeck CE. Patient participation in decision-making about cardiovascular preventive drugs - resistance as agency. Scand J Prim Health Care. 2017;35(3):231–239. 28277056 10.1080/02813432.2017.1288814 PMC5592349

[28] Ahlin K, Billhult A. Lifestyle changes - a continuous, inner struggle for women with type 2 diabetes: a qualitative study. Scand J Prim Health Care. 2012;30(1):41–47. 22324486 10.3109/02813432.2011.654193 PMC3337530

[29] Brobeck E, Odencrants S, Bergh H, et al. Patients' experiences of lifestyle discussions based on motivational interviewing: a qualitative study. BMC Nurs. 2014;13:13. 24904235 10.1186/1472-6955-13-13 PMC4045965

[30] Dallow CB, Anderson J. Using self-efficacy and a transtheoretical model to develop a physical activity intervention for obese women. Am J Health Promot. 2003;17(6):373–381. 12858617 10.4278/0890-1171-17.6.373

[31] Malmstrom M, Sundquist J, Bajekal M, et al. Indices of need and social deprivation for primary health care. Scand J Soc Med. 1998;26(2):124–130. 9658512 10.1177/14034948980260021301

